# Comparison of survival and perioperative outcome of the colonic stent and the transanal decompression tube placement and emergency surgery for left-sided obstructive colorectal cancer: a retrospective multi-center observational study “The CODOMO study”

**DOI:** 10.1007/s00384-020-03806-5

**Published:** 2020-11-27

**Authors:** Shungo Endo, K. Kumamoto, T. Enomoto, K. Koizumi, H. Kato, Y. Saida

**Affiliations:** 1 Department of Coloproctology, Aizu Medical Center, Fukushima Medical University, Aizu-Wakamatsu City, Fukushima 969-3492 Japan; 2 Department of Surgery, Toho University Ohashi Medical Center, Tokyo, Japan; 3 Department of Gastroenterology, Tokyo Metropolitan Cancer Infectious Disease Center Komagome Hospital, Tokyo, Japan; 4 Department of Clinical Laboratory and Endoscopy, Tokyo Women’s Medical University, Medical Center East, Tokyo, Japan

**Keywords:** Obstructive colorectal cancer, Bridge to surgery, Self-expanding metallic stent, Transanal decompression tube, Emergency surgery

## Abstract

**Purpose:**

Advances in endoscopic technology have led to the reevaluation of self-expandable metallic stent (SEMS) placement as a bridge-to-surgery (BTS) in patients with obstructive colorectal cancer. In Japan, after inclusion of SEMS placement as a BTS in the medical insurance coverage in 2012, this procedure has been increasingly performed. However, a transanal decompression tube (TADT) placement has been used as a BTS. We aimed to retrospectively evaluate the optimal strategy for obstructive left-sided colorectal cancer (OLCRC) by comparing SEMS and TADT placement with emergency surgery.

**Methods:**

We included 301 patients with stage II and III OLCRC from 27 institutions. The study patients were divided into Surgery group (emergency surgery, *n* = 103), SEMS group (BTS by SEMS, *n* = 113), and TADT group (BTS by TADT, *n* = 85). We compared the survival and perioperative outcomes of patients in the Surgery group as a standard treatment with those in the SEMS and TADT groups.

**Results:**

The 3-year relapse-free survival rate in patients in the Surgery group was 74.8%, while that in patients in the SEMS group and TADT group were 69.0% (*p* = 0.39) and 55.3% (*p* = 0.006), respectively. The technical success rate was not statistically different, but the clinical success rate was significantly higher in the SEMS group than in the TADT group (*p* = 0.0040). With regard to postoperative complications after curative surgery, the SEMS group had significantly lower of complications (≥ grade 2) than the Surgery group (*p* = 0.022).

**Conclusion:**

Patients who underwent SEMS placement for OLCRC had similar oncological outcomes to patients who underwent emergency surgery.

## Introduction

Colorectal cancer (CRC) is the most common cancer in the gastrointestinal tract in the world [1]. The incidence of obstruction in left-sided CRC was reported to be higher than that in right-sided CRC. Moreover, 8–16% of CRC patients initially present with bowel obstruction, which accounts for 85% of colonic emergencies [1, 2]. Treatment for obstructive left-sided CRC (OLCRC) has been an emergency surgery including stoma creation for colonic decompression [3]. However, the emergency surgical procedure is associated with higher rates of mortality and morbidity compared to elective surgical procedure [4, 5].

In Japan, transanal decompression tube (TADT) has been used as a decompression method to provide a bridge to surgery, which was first reported by Lelcuk et al. in 1985 [5]. Since there is solid stool matter in the dilated colon of patients with colonic obstruction due to left-sided CRC, TADT is not so effective, and management during decompression is complicated. Inevitably, placement of a tube from the anus also has a significant negative impact on the patient’s quality of life (QOL) [6].

As self-expandable metallic stent (SEMS), which was first reported by Dohmoto et al. [7], has been covered by Japanese medical insurance in 2012, emergency operation and colonic stent have become a mainstream treatment for the obstructive CRC instead of TADT. However, “bridge to surgery” (BTS) by colonic stent is not recommended in the European guidelines proposed in 2014. Moreover, this guideline was reviewed and endorsed by the Governing Board of the American Society for Gastrointestinal Endoscopy (ASGE) [8]. We believe that the primary reason for this was due to the poor oncological outcome and many complications associated with stent placement in the cited literature. The Japan Colonic Stent Safe Procedure Research Group (JCSSPRG) has achieved lower complication rate in obstructive CRC by following their mini-guidelines, published on the JCSSPRG website [9]. We retrospectively evaluated the optimum strategy for OLCRC by comparing the oncologic and perioperative outcomes using SEMS and TADT with emergency surgery in Japan.

## Methods

### Study design

A retrospective multi-center observational study was conducted in the JCSSPRG. Twenty-seven institutions were invited to participate in this project. A case report form was used to collect cases from the participating institutions, from August 30, 2017, to July 30, 2019. The medical ethics committee of Fukushima Medical University reviewed and approved the observational study design and decided that the requirement for informed consent was not necessary owing to the observational design of the study. This study was registered in the Japan University Hospital Medical Information Network-Clinical Trials Registry (UMIN000024488).

To disseminate details about the colonic stent procedure to the participating facilities before the start of study, JCSSPRG launched a study group Web site (http://colon-stent.com/), posted the standard procedure as mini-guidelines (brief guidelines for safe placement of colonic stents), and held workshops to discuss a safe procedure for stent placement. The protocol of this study stated that participants would be referred the mini-guidelines. A video of each stent placement procedure was also uploaded to the Web site, accompanied by a written explanation as the characteristics of various stent are quite different. Postoperative complications were defined according to the Clavien-Dindo classification [10].

### Patient selection

**Fig. 1:**
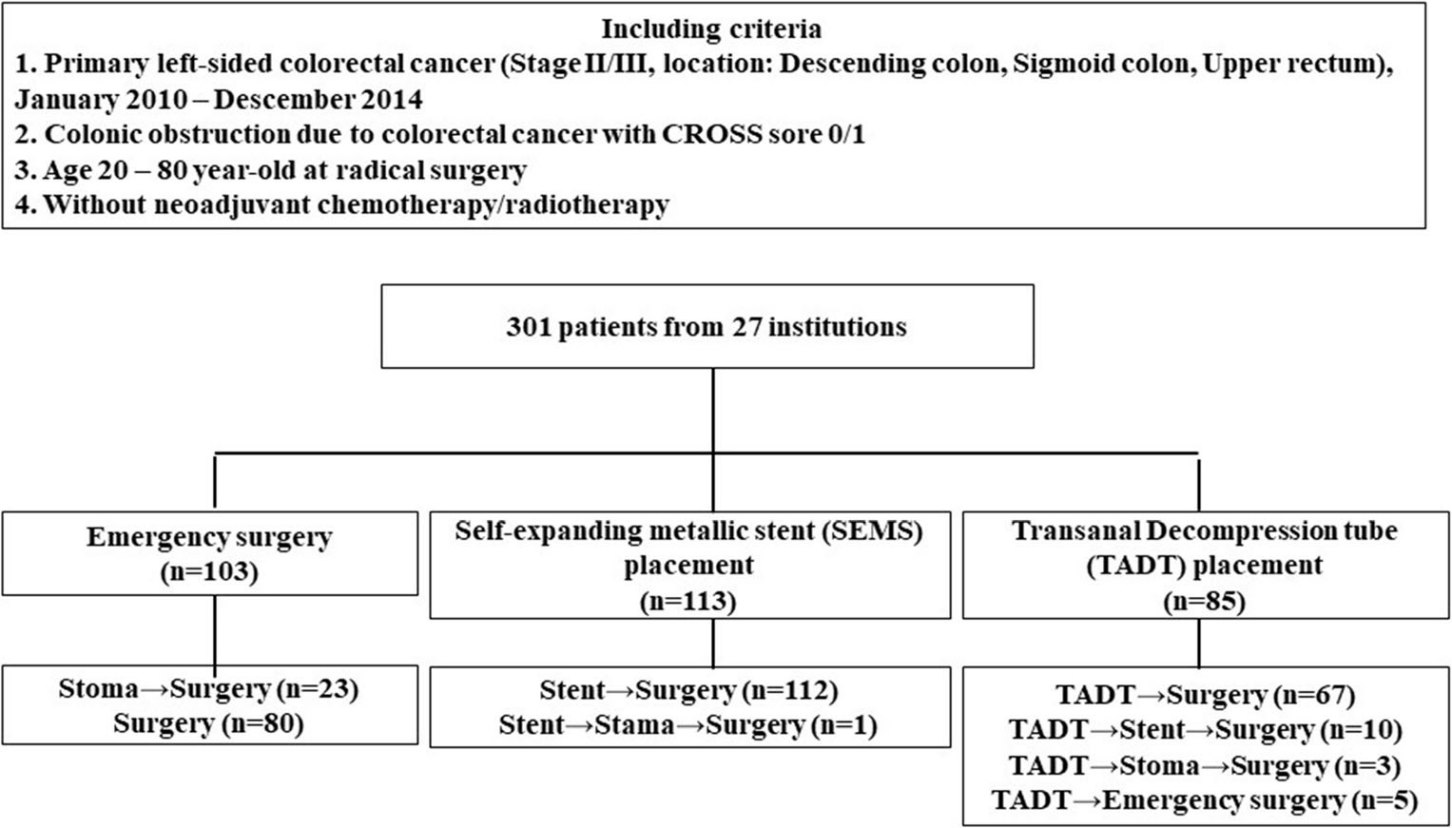
Study design with the colonic obstruction management. CROSS sore, ColoRectal Obstruction Scoring System

The subjects were patients with histologically proven stage II/III left-sided colon or upper rectal cancer with obstruction, who underwent subsequent surgery with curative resection between January 2010 and December 2014. The definition of the obstruction was specified based on the ColoRectal Obstruction Scoring System (CROSS) [11], wherein patient’s oral intake level is assessed as follows: CROSS 0, requiring continuous decompression; CROSS 1, no oral intake; CROSS 2, liquid or enteral nutrient intake; CROSS 3, soft solids, low-residue, and full diet with symptoms of stricture; or CROSS 4, soft solids, low-residue, and full diet without symptoms of stricture. CRC patients with CROSS score 0 and 1 were included in this study. The patient age ranged from 20 to 80 years. Patients treated with neoadjuvant chemotherapy and/or radiotherapy were excluded. Subsequently, 301 patients from 27 institutions met these criteria (Fig. 1). Patients were divided into three groups based on the decompression procedures: The Surgery group with decompression by colostomy or intraoperative decompression during radical surgery (emergency surgery, *n* = 103), the SEMS group using SEMS for BTS (self-expanding metallic stent placement, *n* = 113), and the TADT group with decompression using TADT for BTS (transanal decompression tube placement, *n* = 85). The two-step surgery for curative resection for OLCRC is one of the techniques of bridging to surgery and thought to be standard treatment. Therefore, these cases (23cases, 22.3%) were included in the Surgery group as a standard treatment. We compared the short-term and long-term outcomes of patients with the Surgery group to those with the SEMS and TADT groups, respectively. Since BTS using the TADT is not considered to be the standard treatment, we did not compare the SEMS with TADT groups except for their technical and clinical success rate. Moreover, BTS using the TADT thought going to be replaced by SEMS.

### Endpoints

The primary endpoint was the relapse-free survival (RFS) duration on an intention-to-treat basis. RFS was defined as the time between curative surgery and the first relapse, death from any cause when no evidence of relapse was recorded, or the last date at which the patient was known to be free of disease.

The secondary endpoints were as follows:

1. Technical and clinical success rate of decompression using SEMS and TADT
2. Adverse events during decompression
3. Postoperative complications after curative surgery
4. Temporary/permanent stoma rate after curative surgery
5. Induction rate of adjuvant chemotherapy
6. Decompression period, the length of a hospital stay after curative surgery and a total hospital stay
7. Primary recurrence sites and patterns

### Adjuvant chemotherapy and follow-up

Japanese guidelines for the treatment of CRC [12] indicate that patients with stage III CRC are eligible for postoperative adjuvant chemotherapy; stage II CRC patients with obstruction may be eligible for adjuvant chemotherapy. Recommended therapies are as follows: (1) intravenous fluorouracil and levofolinate, (2) oral uracil and tegafur plus leucovorin, (3) capecitabine, and (4) 5-FU/folic acid combined with oxaliplatin (FOLFOX) 4 or modified FOLFOX6. However, the follow-up procedures and intervals followed local protocols.

### Statistical analysis

Quantitative data were reported as median (range). All statistical analyses were performed using SPSS ver. 25 (IBM, Armonk, NY, USA). The Mann-Whitney *U* test was used to compare continuous variables, and chi-square (Fisher’s exact tests or Pearson’s chi-square test) were used to compare discrete variables. RFS analysis was conducted using the Kaplan–Meier method and the log-rank test to determine significance of the survival curves. *P* values less than 0.05 were considered statistically significant.

## Results

### Patient characteristics

The demographical characteristics of patients are summarized in Table 1. There were no significant differences in age and gender between the Surgery group and the SEMS/TADT group. The performance status (PS) was significantly lower in the SEMS group and the TADT group than the Surgery group (*p* = 0.045, *p* = 0.011, respectively). The ratio of rectal cancer tended to be higher in the TADT group than the Surgery group (*p* = 0.061). No difference was found in the proportion of patients of stage II and III between the Surgery group and the SEMS group, although the rate of stage III was significantly higher in the TADT group than that of the Surgery group (*p* = 0.019). There was no statistically significant difference between the pretreatment serum CEA level of the Surgery group and the SEMS/TADT group.

**Table 1:** Demographical characteristics of the study population

	Surgery group (103 patients)	SEMS group (113 patients)	*P* value (Surgery vs. SEMS)	TADT group (85 patients)		*P* value (Surgery vs. TADT)
	Number of patients (%)	Number of patients (%)		Number of patients %		
Age (years), median (range)	67 (28–80)	69 (48–80)	N.S	69 (52–80)		N.S
Gender			N.S			N.S
Male	64 (62.1)	69 (61.1)		43 (50.6)		
Female	39 (37.9)	44 (38.9)		42 (49.4)		
PS (ECOG)			0.045 (PS 0-1 vs. PS 2-4)			0.011 (PS 0-1 vs. PS 2-4)
0	68 (66.0)	69 (61.1)		47 (55.3)		
1	29 (28.2)	27 (23.9)		22 (25.9)		
2	5 (4.6)	7 (6.2)		10 (11.8)		
3	0	10 (8.8)		4 (4.7)		
4	1 (1.0)	0		2 (2.4)		
Tumor location			N.S (Colon vs. Rectum)			0.061 (Colon vs. Rectum)
Colon	96 (93.1)	106 (93.8)		71 (83.6)		
Descending	25 (24.3)	19 (16.8)		19 (22.4)		
Sigmoid colon	71 (68.9)	87 (77.0)		52 (61.2)		
Rectum	7 (6.8)	7 (6.2)		14 (16.5)		
Stage (TNM)			N.S			0.019
II	63 (61.2)	64 (56.6)		37 (43.5)		
III	40 (38.8)	49 (43.4)		48 (56.5)		
CEA (ng/mL), median (range)	5.7 (1.2–495.3)	5.8 (1.1–174.3)	N.S	7.3 (1.4–111.0)		N.S
Follow up period (months), median (range)	56.2 (1.2–92.1)	46.2 (4.2–78.2)	N.S	48.9 (0.2–93.2)		N.S

### Primary endpoint

**Fig. 2:**
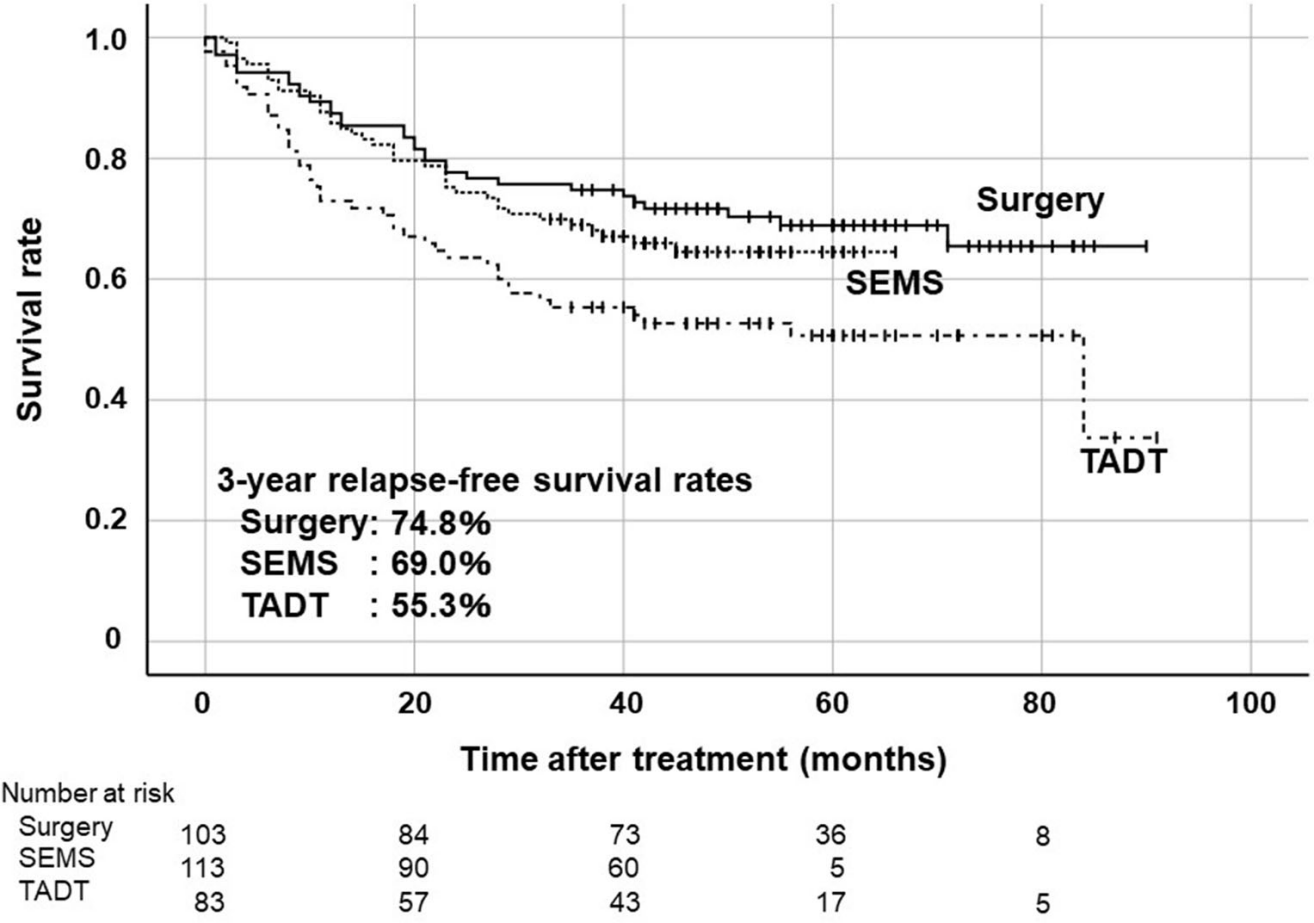
Kaplan-Meier curves of relapse-free survival rates on an intension-to-treat basis in all cases. Surgery vs SEMS: *p* = 0.39, Surgery vs TADT: *p* = 0.006 by log-rank test

**Fig. 3:**
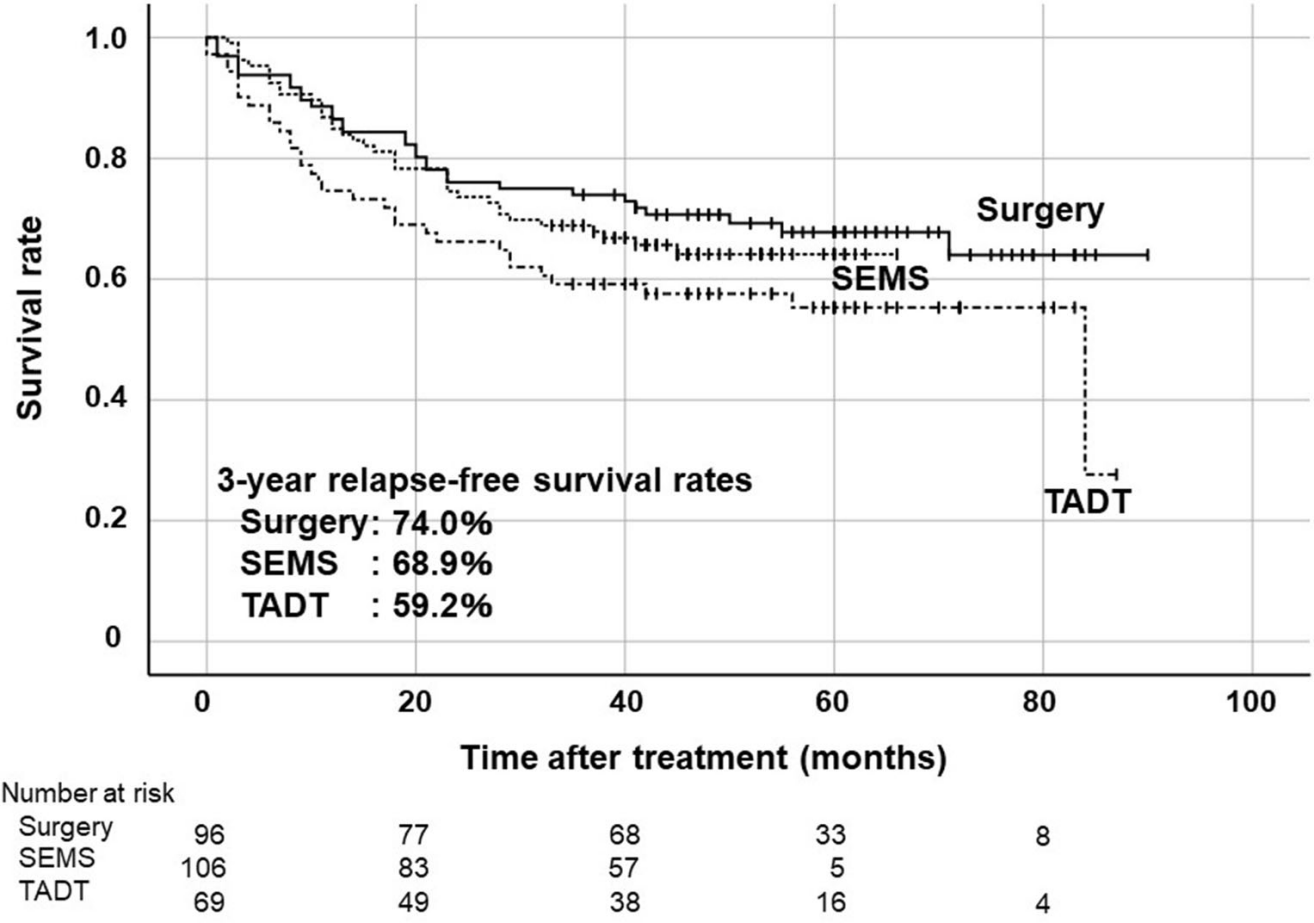
Kaplan-Meier curves of relapse-free survival rates on an intension-to-treat basis in location of descending colon and sigmoid colon. Surgery vs SEMS: *p* = 0.47, Surgery vs TADT: *p* = 0.06 by log-rank test

**Fig. 4:**
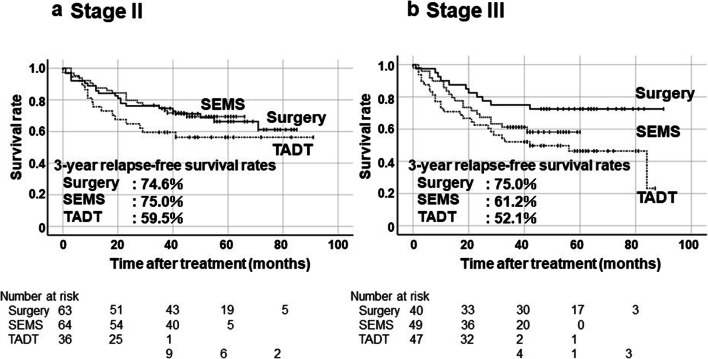
Kaplan-Meier curves of relapse-free survival rates on an intension-to-treat basis in Stage II and Stage III. Surgery vs SEMS: *p* = 0.87, Surgery vs TADT: *p* = 0.23 in Stage II by log-rank test (**a**), Surgery vs SEMS: *p* = 0.16, Surgery vs TADT: *p* = 0.013 in Stage III in log-rank test (**b**)

The 3-year RFS rates were 74.8% in the Surgery group, 69.0% in the SEMS group, and 55.3% in the TADT group (Fig. 2). Although there was no statistically significant difference in RFS rate between the Surgery group and the SEMS group, the 3-year RFS rate of the TADT group was significantly lower than that of the Surgery group (*p* = 0.006). Subgroup analysis was performed with only colon cancer cases because the ratio of rectal cancer was tended to higher in the TADT group as mentioned above (Fig. 3). The 3-year RFS rates of patients with left-sided colon cancer (descending or sigmoid colon cancer) were 74.0% in the Surgery group, 68.9% in the SEMS group and 59.2% in the TADT group. There was no significant difference between the Surgery group and the SEMS group, and the TADT group was relatively lower than the Surgery group (*p* = 0.06). The 3-year RFS rates of patients with stage II were 74.6%, 75.0%, and 59.5% in the Surgery group, SEMS and TADT groups, respectively (Fig. 4a). The 3-year RFS rates of patients with stage III were 75.0%, 61.2%, and 52.1% in the Surgery, SEMS, and TADT groups, respectively (Fig. 4b). No significant difference between the Surgery group and the SEMS/TADT group was observed in patients with stage II. RFS in stage III patients with the TADT group was significantly lower than that in patients with the Surgery group (*p* = 0.013).

### Secondary endpoints

Technical success rate was not statistically different between the SEMS group: (99.1%) and the TADT group (94.1%), but clinical success rate was significantly higher in the SEMS group (97.3%) compared with the TADT group (85.9%) (*p* = 0.004) (Table 2). There was no difference in complication rates during decompression including perforation, migration and re-obstruction between the SEMS group and the TADT group. The number of cases requiring emergency surgery was significantly higher in the TADT group than the SEMS group (*p* = 0.009).

**Table 2:** Technical and clinical success rates and complications during decompression

	SEMS group (113 patients)	TADT group (85 patients)	*P* value
	Number of patients (%)	Number of patients (%)	
Technical success rate	112 (99.1)	80 (94.1)	N.S
Clinical success rate	110 (97.3)	73 (85.9)	0.004
Complications during decompression			N.S
Perforation	2 (1.8)	2 (2.4)	
Migration	0	5 (5.9)	
Re-obstruction	1 (0.9)	0	
Emergency operation during decompression	3 (2.7)	11 (12.9)	0.009

Treatment characteristics of the study population are shown in Table 3. Comparison of the SEMS group and the Surgery group showed that the rate of laparoscopic procedure was significantly higher in the SEMS group (*p* < 0.0001), temporary/permanent stoma rate was significantly lower in the SEMS group (*p* < 0.0001), and final permanent stoma rate was lower in the SEMS group (*p* = 0.008). There was no difference in these rates between the Surgery group and the TADT group. There were no significant differences in depth of invasion between the Surgery group and the SEMS/TADT group, and the ratio of positive lymph node metastasis cases were significantly higher in the TADT group than the Surgery group (*p* = 0.021). The number of harvested lymph nodes was higher in SEMS group than the Surgery group (*p* < 0.001), while there was no difference between Surgery group and TADT group. When the proportion of patients who received adjuvant chemotherapy was compared between stage II and stage III, there were no difference between the treatment groups.

**Table 3:** Treatment characteristics of the study population

	Surgery group	SEMS group	*P* value (Surgery vs. SEMS)	TADT group	*P* value (Surgery vs. TADT)
	(103 patients) Number of patients (%)	(113 patients) Number of patients (%)		(85 patients) Number of patients (%)	
Surgical approach			< 0.0001		< 0.0001
Open	100 (97.1)	57 (50.4)		64 (77.1)	
Laparoscopic	3 (2.9)	56 (49.6)		21 (24.7)	
Stoma					
Temporary/permanent	47 (45.6)	6 (5.3)	< 0.0001	27 (31.8)	N.S
Permanent	11 (10.7)	2 (1.8)	0.008	15 (17.6)	N.S
Depth of invasion (TNM)			N.S		N.S
T2	0	1 (0.9)		0	
T3	58 (56.3)	81 (71.7)		48 (56.5)	
T4a	33 (32.0)	23 (20.4)		29 (34.1)	
T4b	12 (11.7)	8 (7.1)		8 (9.4)	
Lymph node metastasis (TNM)			N.S*		0.021*
N0	63 (61.2)	64 (56.6)		37 (43.5)	
N1	36 (35.0)	35 (31.0)		38 (44.7)	
N2	4 (3.9)	14 (12.4)		10 (11.8)	
Stage (TNM)					
IIA	31 (30.1)	53 (46.9)		16 (19.0)	
IIB	23 (22.3)	9 (8.0)		16 (19.0)	
IIC	9 (8.7)	2 (1.8)		5 (5.9)	
IIIA	0	1 (0.9)		0	
IIIB	36 (35.0)	35 (31.0)		39 (45.9)	
IIIC	4 (3.9)	13 (11.5)		9 (10.6)	
Number of harvested lymph nodes, mean (range)	16 (0–49)	22 (7–106)	< 0.0001	18 (3–80)	N.S
Adjuvant chemotherapy					
Stage II	17 (27.0)	21 (32.8)	N.S	13 (35.1)	N.S
Stage III	30 (75.0)	31 (63.3)	N.S	36 (75.0)	N.S

*Pearson’s chi-square test

The decompression period and the hospitalization are shown in Table 4. In the Surgery group, the decompression period was calculated in patients who underwent radical surgery after stoma creation. The postoperative hospital stay was significantly shorter in the SEMS group and the TADT group than the Surgery group (*p* < 0.0001, *p* = 0.038, respectively). These facts were manifested in the results that the total combined hospital stay for the decompression and postoperative period was significantly shorter in the SEMS group than in the Surgery group (*p* = 0.048).

**Table 4:** Decompression periods and hospital stays

	Surgery group (103 patients)	SEMS group (113 patients)	TADT group (85 patients)
Decompression period (day), mean (range)	57.0 (1–396)*	17.0 (2–84)	10.0 (0–43)
Hospital stay after curative surgery (day), mean (range)	16.0 (7–225)	11.0 (5–62)**	14.0 (6–167)**
Total hospital stay (day), mean (range)	28.0 (7–225)	23.0 (8–81)^†^	28.0 (10–171)

*Decompression period of the Surgery group was calculated by cases decompression of stoma before curative surgery

**Hospital stay after curative surgery were shorter in the SEMS group and the TADT group than the Surgery group (*p* < 0.0001, *p* = 0.038, respectively)

^†^Total hospital stay was shorter in the SEMS group than the Surgery group (*p* = 0.048)

Postoperative complications after curative surgery were classified using the Clavien-Dindo classification (Table 5). Total number of complications (≥ grade 2) after curative surgery were significantly lower in the SEMS group than the Surgery group (*p* = 0.022), while there was no statistical difference between the Surgery group and the TADT group. The occurrence of postoperative ileus (≥ grade 3) was significantly higher in the SEMS group than the Surgery group (*p* = 0.03).

**Table 5:** Postoperative complications after curative surgery

	Surgery group (103 patients)	SEMS group (113 patients)	TADT group (85 patients)
	Number of patients	Number of patients	Number of patients
Complication (≥G2) (yes:no)	36:67	23:90*	25:60
Superficial incisional SSI (G2:≥G3)	8:0	3:0	5:0
Deep incisional SSI (G2:≥G3)	0:1		
Space/organ SSI (G2:≥G3)	1:0	1:0	1:1
Anastomotic leakage (G2:≥G3)	4:6	2:5	2:2
Anastomotic hemorrhage (G2:≥G3)		0:2	
Anastomotic stenosis (G2:≥G3)		0:1	
Ileus (G2:≥G3)	8:0	1:6**	4:1
Necrotic/ischemic enteritis (G2:≥G3)	0:1	0:1	2:0
Pneumonia (G2:≥G3)	3:0		0:1
Incisional abdominal hernia (G2:≥G3)	0:1	0:1	
Thrombosis (G2:≥G3)	0:1		
Cholecystitis (G2:≥G3)	1:1	0:1	2:0
Sepsis (G2:≥G3)			0:1
Renal failure (G2:≥G3)	1:0		
Arrhythmia (G2:≥ G3)	0:1		
Gastrointestinal perforation (G2:≥G3)			0:1
Others (G2:≥G3)	1:0	4:0	2:1

*All complications (≥G2) were lower in the SEMS group than the Surgery group (*p* = 0.022)

**Ileus (≥G3) was higher in the SEMS group than the Surgery group (*p* = 0.030)

The sites of recurrence are shown in Table 6. There was no statistically difference in total recurrence rate between the SEMS group and the Surgery group (*p* = 0.088), while in the TADT group was significantly higher than that in the Surgery group (*p* = 0.006). There was no statistically difference in the peritoneal recurrence rates between three groups. The hematogenous metastases, to the liver, lung and brain, were found in 11 cases (10.7%) of the Surgery group, 21 cases (18.6%) of the SEMS group, and 17 cases (20.0%) of the TADT group. Although the frequency of the hematogenous metastases in the TADT group was more common than that in the Surgery group, the difference was not statistically significant.

**Table 6:** Sites of recurrence

	Surgery group (103 patients)	SEMS group (113 patients)	TADT group (85 patients)
	Number of patients (%)	Number of patients (%)	Number of patients (%)
Liver	8 (7.8)	16 (14.2)	9 (10.6)
Lung	2 (1.9)	5 (4.4)	11 (12.9)
Peritoneum	4 (3.9)	6 (5.3)	8 (9.4)
Lymph node	3 (2.9)	6 (5.3)	2 (2.4)
Local	4 (3.9)	5 (4.4)	4 (4.7)
Brain	1 (1.0)		
Liver+lung			3
Liver+peritoneum	1	1	
Lung+lymph node			1
Peritoneum+lymph node+local		1	
Total	21 (20.4)	35 (31.0)	33 (38.8)*

*Total recurrence rate was higher in the TADT group than in the Surgery group (*p* = 0.006, OR 0.470, 95% Conf. int. 0.211, 0.772)

## Discussion

In the present study, we clarified that short-term and long-term outcomes of patients with SEMS placement for OLCRC were acceptable when compared with those of patients who underwent emergency surgical procedure. SEMS placement as BTS for curative treatment of obstructive CRC was not recommended as a standard treatment in the European Society of Gastrointestinal Endoscopy (ESGE) guidelines in 2014 [8]. This guideline was based on studies that reported a lower technical success rate of SEMS insertion for a small number of cases about 10 years earlier [13, 14]. However, after the ESGE guideline, many studies, including meta-analyses, have investigated long-term outcomes following SEMS placement compared with emergency resection. According to these reports, no significant survival difference was observed between treatment groups. Most of these studies were retrospective and underpowered, they seldom had recurrence and survival as the primary outcome measures, and follow-up period was often relatively short. Subsequently, reports from Japan [15], South Korea [16], Italy [17], Netherlands [18], and a meta-analysis on short-term outcome [19], showed good success rates of SEMS placement. Another meta-analysis [20] reported that patients with SEMS placement were not inferior to those with emergency surgery in terms of short-term and long-term outcomes. In view of new evidences, the ESGE Guideline was up-dated in April 2020 and recommended the use of SEMS in the treatment of malignant colonic obstruction [21]. Further, ESGE also recommends that colonic stenting should be performed or directly supervised by an operator who has competence in both colonoscopy and fluoroscopic techniques and who performs colonic stenting on a regular basis. Our study was inspired by these results and our results were in accordance with the guidelines.

In this study, we analyzed RFS as a primary endpoint. The SEMS placement as BTS with curative intent in OLCRC was not associated with impaired long-term oncologic outcomes when compared to the Surgery group, but the TADT group had poor outcomes. The 3-year RFS rate in patients with the SEMS group (69.0%) was not statistically significant compared to those with the Surgery group (74.8%). A similar study from Korea, comparing the emergency surgery and the BTS by SEMS reported that the 5-year disease-free survival (DFS) of stage II and III was 51.6% for emergency surgery and 63.3% for SEMS without statistical significance [17]. However, another similar study in Spain reported that 5-year progression-free survival of stage III was 69.7% for SEMS and 30.0% for emergency surgery with significant difference [22]. Meta-analysis reported that stent placement before elective surgery did not adversely affect overall survival and disease-free survival, and there was no significant difference between the randomized and observational studies [23], and ESCO trial from Italy [18] also reported no difference in DFS between the emergency surgery and the BTS by SEMS placement. In the Dutch study [19], when the enrolled cases with curative resection were analyzed, the 3-year DFS was 52.6% in the emergency surgery and 58.8% in the BTS using SEMS, showing no difference in long-term oncological outcomes. Our results were in accordance with these data. On the other hand, the RFS rate in patients with the TADT group (59.2%) was significantly lower than that in patients with the Surgery group (74.8%). This result might be associated with tumor locations and stages. Comparing the patient characteristics, the ratio of patients with upper rectal cancer in the TADT group was approximately twice as higher than that in the Surgery group. It may likely explain the poor prognosis in the TADT group. A subgroup analysis was then performed to exclude upper rectal cancer. As a result, RFS rate of the TADT group tended to be poorer than that of the Surgery group. The ratio of patients with Stage III in the TADT group was significantly higher than that in the Surgery group. There was no significant difference in RFS between each treatment groups in Stage II patients, but RFS rate of Stage III patients in the TADT group was significantly poorer than that in Surgery group. It can be also explained by the poor prognosis in the TADT group. In the present study, induction rates of adjuvant chemotherapy were not different between each treatment groups, although a lower induction rate has been expected in the Surgery group due to postoperative complications. There was also no difference in induction rates of adjuvant chemotherapy in the reports from Korea and the Netherlands [17, 19].

The patients were enrolled from twenty-seven Japanese centers in this study. These data may approximate real-world data, although there was some variability in treatment strategies. The demographical characteristics show that the median follow-up period was 46.2 months of the SEMS group, which was equal or longer than that reported from the Netherlands [19] and cannot be considered to be of short duration. The patients of the Surgery group had better PS than that of the SEMS group and the TADT group. This seems to be a reasonable result for a retrospective observational study. A previous study [19] had reported that emergency surgery was performed to younger patients and SEMS to older patients, but this could have been due to a difference in PS rather than age, in this study. The proportion of upper rectum cases tended to be higher in the TADT group than other groups. Emergency surgery was less preferred for upper rectum because of longer operative time, the higher incidence of postoperative complications especially anastomotic leakage. The SEMS group had less upper rectum cases as it is associated with complications such as pain, tenesmus, incontinence, and stent migration, and fear that the inflammation of the anal side of rectum by SEMS would make radical surgery difficult [21, 24, 25].

A number of meta-analyses have reported favorable short-term results for SEMS compared to emergency surgery in terms of temporary/permanent stoma rates, primary anastomosis rates, and postoperative complications, including anastomotic leakage [16, 20, 26, 27]. The success rate of stent placement was low causing poor prognosis for BTS by stent placement [28], but in recent years, the success rate had improved [29]. A report from the Netherlands [19] found no difference in the long-term prognosis of emergency surgery and BTS by SEMS and described that SEMS had technical success of 87.5% with clinical success of 81.1%. In the present study, the technical and clinical success rates for SEMS placement were 99.1% and 97.3%, respectively, and these were likely to be improved because of the JCSSPRG mini-guidelines. Although the results of this study may not support the association between stent related microperforation and dissemination of tumor cells, our findings emphasize that SEMS placement as a BTS may be safely performed with experienced endoscopists. An epidemiological study using the Japanese Diagnosis Procedure Combination database showed that SEMS patients are inferior to emergency surgery patients in overall survival rate [30]. However, in that study, the success rate of SEMS placement was unknown, it was evaluated by overall survival rate, and it had a short follow-up period of 14.9 months for SEMS and 14.7 months for emergency surgery, which may have influenced the results.

TADT was one of decompression methods of BTS for OLCRC since the 1990s because SEMS was not being covered by insurance system in Japan. But there has been no consensus on the evaluation of TADT, and it is still controversial [6, 31]. Although TADT is considered to have a negative impact on QOL, the expenses of TADT is much lower than that of SEMS. For these reasons, TADT has been used as a procedure of BTS in some Asian countries [32, 33]. In a meta-analysis comparing the success rates, both technical and clinical success rates were significantly better for SEMS than for TADT [6]. This meta-analysis also reported significantly better tumor resection rates, primary anastomosis rates, stoma rates for SEMS, and trend to benefit in the SEMS than in the TADT for complications related to decompression including perforation (though the difference was not significant). Postoperative complications rates, including anastomotic leakage, surgical site infection, and ileus, were similar. The postoperative hospital stay was shorter with SEMS, but the difference did not reach significance. In terms of long-term outcomes, the 5-year DFS of stage II and III was reported to be 72.2% for SEMS and 52.0% for TADT [33].

Our result suggested that total recurrence rate in the SEMS group tended to be higher than that in the Surgery group. There are few detailed reports on the sites of recurrence. There was no difference between the SEMS and the emergency surgery in locoregional recurrence, including peritoneal metastasis, in reports where recurrence sites were described [17, 19]. This may be due to the low rate of complications such as perforation during stent placement. The frequency of hematogenous metastases compiled from reports of OLCRC did not differ, being 26.1% by SEMS and 32.2% by emergency surgery, respectively [19]. This is similar to the results of our study. A study comparing recurrence rates in SEMS and TADT [21] reported no significant difference in locoregional spread with rate of 1.9% and 13.0%, respectively, or hematogenous spread with rates of 20.8% and 21.7%, respectively, but this study had small sample size (76 cases), and it is possible that the assessment of locoregional recurrence could have changed if the number of cases increased.

This study has several limitations that must be taken into account. First, even though this study is a multicenter study, it is a retrospective study. The indication for SEMS insertion before a radical surgery was not strictly like that in other retrospective studies. Although considering the obstacles, such as the requirement of large sample size, emergency setting, and the difficulty in technical standardization, pursuing a multicenter RCT on this topic seems difficult, and JCSSPRG has been currently conducting RCT, the results of which are awaited. Second, the procedure for the SEMS placement was based on mini-guidelines but no procedure was defined for the TADT placement. Third, type of SEMS (lumen diameter, etc.) used for BTS in this study was not determined. Fourth, only patients who underwent colonic resection were included in this study. Thus, patients who died as a consequence of SEMS placement and TADT placement remained beyond the scope of the present study. However, as no postoperative deaths after SEMS placement have been reported in any previous reporting on SEMS as BTS, the influence of this shortcoming is expected to be negligible. In this study, long-term outcome was assessed by the RFS as a primary endpoint. Due to the significant advances in therapeutic chemotherapy in recent years, we believe that there would be no difference in long-term outcome based on the overall survival. There is also an option to evaluate the propensity score matching method, but due to the small number of cases enrolled, we evaluated all cases included in this study.

## Conclusion

This study suggests that SEMS placement followed by surgery, has no adverse influence in terms of the patient relapse-free survival, compared with emergency surgery. Additionally, total number of complications after curative surgery were significantly lower in the SEMS group than the Surgery group. SEMS placement performed by experienced endoscopist, can be a treatment option for OLCRC as well as emergency surgery.
